# ^99m^Tc-labeled iRGD for single-positron emission computed tomography imaging of triple-negative breast cancer

**DOI:** 10.3389/fbioe.2022.1001899

**Published:** 2022-09-19

**Authors:** Buhui Yu, Hongxing Su, Lingzhou Zhao, Jiqin Yang, Meilin Zhu, Jinhua Zhao

**Affiliations:** ^1^ Department of Nuclear Medicine, Shanghai General Hospital, Shanghai Jiao Tong University School of Medicine, Shanghai, China; ^2^ Department of Nuclear Medicine, General Hospital of Ningxia Medical University, Yinchuan, Ningxia, China; ^3^ School of Basic Medical Sciences, Ningxia Medical University, Yinchuan, Ningxia, China

**Keywords:** triple-negative breast cancer, iRGD, neuropilin-1, integrin αvβ3, SPECT imaging

## Abstract

Triple-negative breast cancer (TNBC) is the most aggressive breast cancer subtype, with a high mortality rate. One of the main reasons for this poor prognosis is the failure of a specific diagnosis. As a tumor-homing and penetrating peptide, iRGD has not only the properties of binding to neuropilin-1 and integrin αvβ3 but also internalizing into TNBC cells. In this study, we designed and prepared ^99m^Tc-labeled iRGD (^99m^Tc-HYNIC-iRGD) as a single-positron emission computed tomography (SPECT) imaging probe and investigated its feasibility for the targeted diagnosis of TNBC. The results showed that the iRGD peptide had acceptable biocompatibility within the studied concentration range and could specifically bind to TNBC cells *in vitro*. The ^99m^Tc-HYNIC-iRGD was readily prepared with high radiochemical purity and stability. SPECT imaging of ^99m^Tc-HYNIC-iRGD in a TNBC tumor-bearing mouse model showed obvious tumor accumulation with rapid blood clearance and favorable biodistribution. Our findings indicate that this active-targeted strategy has great potential to be developed as a novel tool for TNBC imaging.

## Introduction

Triple-negative breast cancer (TNBC) is a subtype of breast cancer characterized by a lack of estrogen receptors, progesterone receptors, and human epithelial growth factor receptor 2, accounting for approximately 20% of all breast cancers (Yin et al., 2020). Compared with other subtypes of breast cancer, patients with TNBC have lower survival rates and higher rates of tumor recurrence and metastasis (Garrido-Castro et al., 2019; Yin et al., 2020). A good prognosis of breast cancer is closely related to early diagnosis and effective treatment (Li et al., 2017). However, TNBC lacks common tumor biomarkers of breast cancer, leading to ineffective molecular imaging and targeted therapy (Won and Spruck, 2020). Therefore, achieving sensitive detection in the early TNBC stages continues to gain increased attention. One strategy is to develop novel diagnostic methods based on radionuclide molecular imaging that can provide accurate and sensitive detection of TNBC (Liu et al., 2016; Song et al., 2019; Cavaliere et al., 2021).

An RGD-containing peptide discovered by phage screening, iRGD (CRGDK/RGPD/EC), is highly desirable for improving the targeting ability of imaging agents and enhancing their accumulation in tumors (Sugahara et al., 2009; Houdong; Zuo 2019). It homes to tumors by binding to integrin αvβ3 or αvβ5 through the RGD motif, which is generally overexpressed in tumor cells and tumor neovascular endothelial cells but not in normal tissues. After binding to the cell surface, iRGD is proteolytically cleaved to CRGDK/R, which activates the neuropilin-1 (NRP-1) dependent C-end rule (CendR) sequence (R/KXXR/K) internalization pathway (Teesalu et al., 2009; Desgrosellier and Cheresh, 2010; Sugahara et al., 2010). The excellent tumor targeting and penetrating ability of iRGD enables it to overcome the abundant fibrous connective tissue barrier and high interstitial fluid pressure in the tumor and to penetrate deep into the tumor tissue (Teesalu et al., 2013; Ruoslahti 2017). Based on receptor overexpression in cancers, radiolabeled peptides provide a promising research area in tumor imaging and therapy and can be optimized to be considered as personalized medicine strategies (Rezazadeh and Sadeghzadeh, 2019; Mohtavinejad et al., 2020). TNBC highly expresses integrins and NRP-1, providing conditions for the application of iRGD to TNBC for radionuclide imaging.

In a previous study, iRGD was used as a targeting agent in drug delivery systems. After radiolabeling with. ^111^In, the drug delivery process can be monitored by single-positron emission computed tomography (SPECT) imaging (Wang et al., 2015). Recently, iRGD was radiolabeled with ^68^Ga and showed good targeting ability in melanoma cells (Satpati et al., 2020). These studies showed the potential of iRGD for radionuclide imaging, but studies related to direct radiolabeling of iRGD for tumor SPECT imaging are still lacking, especially research focusing on the application of ^99m^Tc-labeled iRGD for TNBC diagnosis. In this study, we designed and prepared ^99m^Tc-labeled iRGD as a novel SPECT probe for TNBC imaging and investigated its imaging performance in a tumor-bearing mouse model. The cyclic iRGD peptide has flexible structural conformations because it contains a proteolytic cleavage recognition site that can be cleaved to produce CRGDK. Hence, the site of iRGD modified with a bifunctional chelator hydrazinonicotinic acid (HYNIC) was the N-terminus of CRGDK. Our data showed that ^99m^Tc-HYNIC-iRGD could be readily prepared with high radiochemical purity (RCP) and excellent stability *in vitro*. More importantly, ^99m^Tc-HYNIC-iRGD showed favorable biodistribution, fast blood clearance, and obvious tumor accumulation in TNBC mouse models. These results suggest the considerable potential of ^99m^Tc-HYNIC-iRGD as a novel SPECT probe for TNBC imaging.

## Materials and methods

### Materials

CG_7_C and iRGE peptides were used as control peptides for different purposes (Sugahara et al., 2009). The amino acid sequence of iRGE was CRGEKGPDC, and as a non-integrin-binding variant with potential CendR motif, it was used to validate the significance of the RGD motif in targeting TNBC. CG_7_C is a polyglycine control peptide that has the same number of base residues as iRGD but does not bind integrins or NRP-1 and acts as a negative control. The iRGD, iRGE, and CG_7_C were manufactured by Synpeptide Co., Ltd. (Shanghai, China). During the synthesis process, fluorescein isothiocyanate (FITC) and HYNIC were modified at the N-termini of these peptides for cell experiments and ^99m^Tc radiolabeling, respectively. Na^99m^TcO_4_ solution was purchased from Shanghai Atom Kexing Pharmaceutical Co., Ltd. (Shanghai, China). Tricine, ethylenediamine-N, N′-diacetic acid (EDDA), SnCl_2_, cell counting kit-8 (CCK-8), fetal bovine serum (FBS), and RPMI-1640 medium were purchased from Shanghai Dobio Co., Ltd. (Shanghai, China). Other chemicals and solvents were supplied by Sinopharm Chemical Reagent Co. Ltd. (Shanghai, China).

### Cells and animals

Murine breast cancer cells 4T1 were purchased from Procell Life Science and Technology Co., Ltd. (Wuhan, China) and maintained in RPMI-1640 supplemented with 10% FBS. Four-week-old female BALB/c nude mice (18–20 g) and healthy ICR mice (20–22 g) were purchased from Shanghai Laboratory Animal Center of the Chinese Academy of Sciences (Shanghai, China). To establish breast cancer models, four-week-old female BALB/c nude mice were subcutaneously injected in their right-side flanks with 5 × 10^6^ 4T1 cells suspended in 100 μl of PBS. When the tumor diameter reached 0.8–1.2 cm, the mice were used for animal experiments. All animal experiments were repeated three times and performed according to the protocols established by the Ethics Committee of Shanghai General Hospital.

### Synthesis of ^99m^Tc-labeled peptides and quality control

HYNIC-iRGD, HYNIC-iRGE, and HYNIC-CG_7_C were labeled with ^99m^Tc according to previously published procedures in the literature (Bohn et al., 2013; Xu et al., 2017; Gong et al., 2022). In brief, a mixture of 50 μl HYNIC-modified peptide (1 mg/ml in water), 0.5 ml EDDA solution (20 mg/ml in 0.1 M NaOH), 0.5 ml tricine solution (40 mg/ml in 0.2 M PBS, pH = 6.0), 1 ml Na^99m^TcO4 solution (50 mCi/ml), and 50 μl SnCl_2_ solution (1 mg/ml in 0.1 M HCl) were heated at 100°C for 15 min. After cooling to room temperature, the mixture was analyzed using instant thin-layer chromatography (TLC) and radio-high-performance liquid chromatography (radio-HPLC). The ^99m^Tc-labeled peptides were characterized using an Agilent 1260 HPLC system (Agilent Technologies, United States) equipped with a UV-vis detector (*λ* = 220 nm) and a radioactive flow detector (BioScan, United States). A SunFire C18 column (5 μm, 4.6 × 250 mm, Waters, Japan) was used at a flow rate of 1 ml/min with the following gradient method: 0.1% Trifluoroacetic acid in H_2_O and CH_3_CN (0–20 min, 10%–35% CH_3_CN) for iRGD and iRGE, and 0.1% trifluoroacetic acid in H_2_O and CH_3_CN (0–20 min, 25%–60% CH_3_CN) for CG_7_C. Their RCPs were determined by radio-HPLC and rapidly analyzed by TLC in a system consisting of silica gel 60 F254 TLC plates (Merck, Germany) and 50% acetonitrile as mobile phase. To assess stability *in vitro*, the formed ^99m^Tc-labeled peptides were incubated sequentially in two solutions as follows: 1) PBS (0.1 M, pH = 7.4) and cysteine solution (100-fold molar excess over the peptides) at room temperature, and 2) FBS at 37°C. Their RCPs were tested using the TLC method described above within 6 h.

### Cytotoxicity assay

To assess the potential cytotoxic effects of iRGD, iRGE, and CG_7_C, cell proliferation assays were performed using the CCK-8 assay. The 4T1 cells (1 × 10^4^ per well) were seeded in 96-well plates in 100 μl of RPMI-1640 medium with 10% FBS and incubated for 24 h at 37°C and 5% CO_2_. The medium was replaced with 100 μl of fresh medium containing different concentrations of iRGD, iRGE, or CG_7_C (0.1, 1, 5, 10, 20, or 50 μM) and incubated for another 24 h. Next, 10 μl of CCK-8 was added to the wells. After 2 h of incubation, absorbance at 450 nm was measured using a Varioskan Flash multimode microplate reader (Thermo Fisher Scientific, Waltham, MA, United States). Relative cell viability was determined by comparison with control wells treated with fresh medium.

### Confocal microscopy and flow cytometry analysis

The targeting ability of iRGD to 4T1 cells was assessed using confocal microscopy. Briefly, 4T1 cells (2 × 10^5^ cells) were seeded into glass-bottom dishes in 2 ml of RPMI-1640 medium. After 24 h of incubation, the medium was replaced with 2 ml of serum-free medium containing iRGD-FITC, iRGE-FITC, or CG_7_C-FITC at a concentration of 10 μM for 4 h. The control group was treated with drug-free medium at an equal volume of PBS. Then the cells were washed thrice with PBS, followed by fixation with 4% paraformaldehyde for 20 min. The nuclei were then stained with 200 μl of 1 μg/ml 6-Diamidino-2-phenylindole (DAPI) for 5 min and washed thrice with PBS. Fluorescence images were acquired using a Leica SP8 laser confocal microscope (Leica, Wetzlar, Germany).

To quantitatively analyze the specificity of iRGD towards tumor cells, 4T1 cells were cultured in 6-well plates at density of 2 × 10^5^ cells/well in 2 ml of medium and cultured for 24 h. The cells were then washed twice with PBS to remove the remnant medium and incubated with serum-free medium containing iRGD-FITC, iRGE-FITC, or CG_7_C-FITC at a concentration of 10 μM. Cells treated with drug-free medium containing an equal volume of PBS were used as controls. After 4 h of incubation, the cells were trypsinized and centrifuged at 1,000 rpm for 3 min, washed, and resuspended in 1 ml PBS. FITC-positive cells were analyzed in the FL1 fluorescence channel using a BD AccuriTM C6 flow cytometer (BD Biosciences, Franklin Lakes, NJ, United States). A minimum of 10,000 events were recorded for each sample.

### *Ex vivo* fluorescent imaging

The *in vivo* targeting ability of the iRGD was evaluated using fluorescence imaging. Briefly, 4T1 tumor-bearing mice were randomly divided into three groups (three mice per group). After anesthetization with pentobarbital sodium (40 mg/kg), the tumor-bearing mice were intravenously administrated with 150 μl of iRGD-FITC, iRGE-FITC, or CG_7_C-FITC at a concentration of 1 mg/ml, then sacrificed at 1 h post-injection to collect the tumors and main organs. The samples were imaged using a fluorescence imaging system (IVIS Lumina Series III, PerkinElmer, United States) with excitation and emission wavelengths of 535 and 580 nm, respectively.

### *In vivo* single-positron emission computed tomography imaging and immunohistochemistry

The 4T1 tumor-bearing mice were randomly divided into three groups (three mice per group). After anesthetization with pentobarbital sodium (40 mg/kg), 200 μl of ^99m^Tc-HYNIC-iRGD, ^99m^Tc-HYNIC-iRGE, or ^99m^Tc-HYNIC-CG_7_C ([^99m^Tc] = 10 mCi/ml) was injected *via* the tail vein. SPECT images were acquired at 0.5, 1, 2, and 4 h post-injection using a SPECT imaging system equipped with a Xeleris 2.0 workstation and low-energy general-purpose collimators (Infinia, Denver, CO, United States).

After SPECT imaging, muscle and tumor tissue samples were isolated from the mice, fixed in 10% formalin at room temperature, embedded in paraffin, and processed into 4 µm continuous sections. Tissue slices were dewaxed using discontinuous concentrations of ethanol and immersed in ethylenediaminetetraacetic acid antigen retrieval buffer (pH = 9.0) for microwave antigen retrieval. Then the slices were blocked to inhibit endogenous peroxidase, and 3% BSA was added dropwise at room temperature. These slices were then incubated with anti-integrin αvβ3 (1:50) and anti-neuropilin-1 (1:100) antibodies at 4°C overnight. Horseradish peroxidase conjugated goat anti-rabbit antibody was used as the secondary antibody to stain the slices at room temperature for 50°min, followed by staining with DAB. Cell nuclei were stained blue with hematoxylin. Samples were then dehydrated, cleared with xylene, mounted, and photographed.

### Biodistribution

The 4T1 tumor-bearing mice were intravenously injected with ^99m^Tc-HYNIC-iRGD, ^99m^Tc-HYNIC-iRGE, or ^99m^Tc-HYNIC-CG_7_C (200 μl, 20 μCi) to determine biodistribution properties. The mice were sacrificed at 0.5, 1, 2, and 4 h post-injection, and the major organs, including the liver, spleen, kidneys, heart, lung, stomach, intestine, muscle, and tumor, were collected and weighed. The radioactivity counts of all samples were measured using a γ-counter (CAPINTEC, United States) and expressed as counts per minute after correction for decay. The results are shown as the percentage of injection dose/gram (%ID/g) of wet tissue for each time point in each group.

### Pharmacokinetics

The pharmacokinetic profiles of ^99m^Tc-HYNIC-iRGD and ^99m^Tc-HYNIC-iRGE were evaluated in healthy ICR mice. Each mouse was intravenously injected with ^99m^Tc-HYNIC-iRGD or ^99m^Tc-HYNIC-iRGE at a dose of 20 μCi in 200 μl PBS solution. Blood samples (100 μl) from each mouse were immediately collected and weighed at designated times (1, 2, 5, 15, 30, 60, 120, and 180 min), and the radioactivity was measured using a γ-counter to calculate the %ID/g. Moreover, the pharmacokinetic data were analyzed by DAS 2.0 (Shanghai, China) using a two-compartment model to calculate the half-lives of ^99m^Tc-HYNIC-iRGD and ^99m^Tc-HYNIC-iRGE in blood.

### Statistical analysis

Data were expressed as means ± standard deviation (SD). Statistical data analysis was performed by one-way analysis of variance with *p* < 0.05 as the minimal level of significance. The data were described as follows: ∗ < 0.05, ∗∗ < 0.01, and ∗∗∗ < 0.001.

## Results

### Radiolabeling and quality control

**FIGURE 1:**
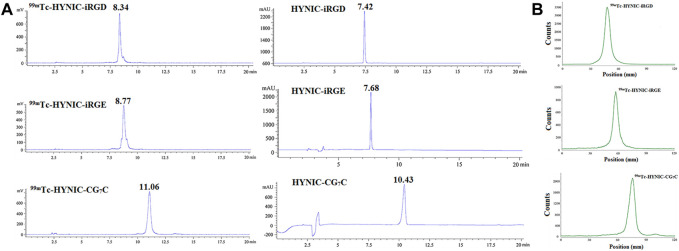
**(A)** Radio-HPLC and **(B)** TLC results of ^99m^Tc-HYNIC-iRGD, ^99m^Tc-HYNIC-iRGE, and ^99m^Tc-HYNIC-CG_7_C.C.

HYNIC-modified iRGD, iRGE, and CG_7_C could be effectively radiolabeled with ^99m^Tc in one pot using tricine and EDDA as co-ligands. The resulting ^99m^Tc-HYNIC-iRGD, ^99m^Tc-HYNIC-iRGE, and ^99m^Tc-HYNIC-CG_7_C were characterized by radio-HPLC. As depicted in Figure 1A, single radioactive peaks of ^99m^Tc-HYNIC-iRGD, ^99m^Tc-HYNIC-iRGE, and ^99m^Tc-HYNIC-CG_7_C were observed with retention times of 8.34°min, 8.77°min, and 11.06°min, respectively, matching up with the corresponding HYNIC-iRGD (7.42 min), HYNIC-iRGE (7.68 min) and HYNIC-CG_7_C (10.43 min). Their RCPs were calculated to be greater than 95%, suggesting that the formed ^99m^Tc-labeled peptides could be used for *in vitro* and *in vivo* experiments without further purification. Moreover, RCPs could be rapidly estimated using TLC. The data showed that colloidal ^99m^Tc and Na^99m^TcO_4_ had retention factors (R_f_) of 0–0.2 and 0.8–1.0, respectively, while ^99m^Tc-HYNIC-iRGD, ^99m^Tc-HYNIC-iRGE, or ^99m^Tc-HYNIC-CG_7_C displayed a R_f_ of 0.4–0.6 (Figure 1B). Furthermore, no obvious changes in RCPs were found in PBS and cysteine solution at room temperature or FBS at 37°C within 6°h, suggesting high stability of ^99m^Tc-HYNIC-iRGD, ^99m^Tc-HYNIC-iRG, and ^99m^Tc-HYNIC-CG_7_C *in vitro*.

### Cytotoxicity

**FIGURE 2:**
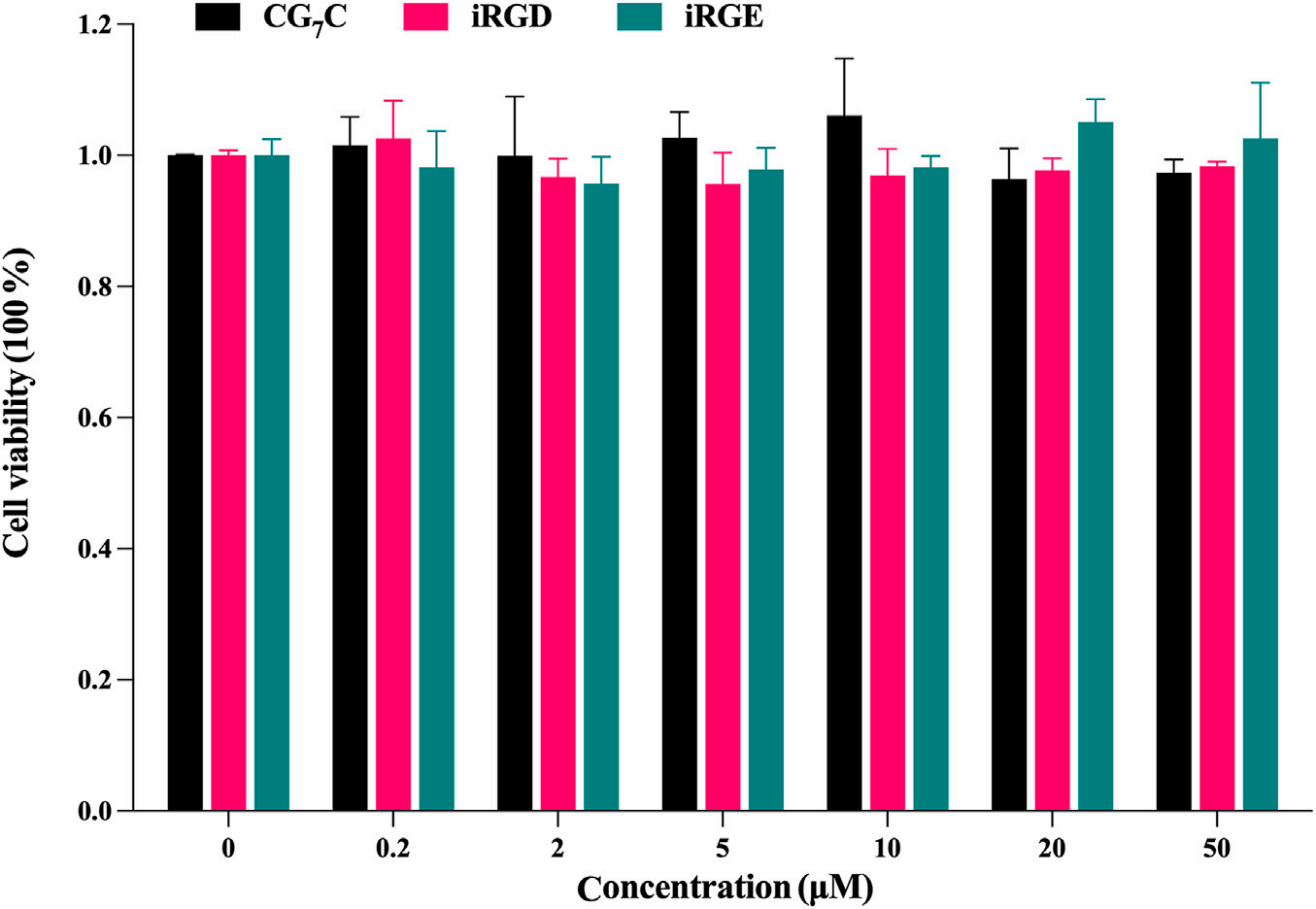
Cytotoxic effects of CG_7_C, iRGD, and iRGE on 4T1 cells at concentrations from 0 to 50 μM after incubation for 24 h.

The potential cytotoxicities of iRGD, iRGE, and CG_7_C to 4T1 cells were tested using CCK-8 assay. After treatment with different concentrations for 24°h, the cell viabilities showed no obvious change between control peptides and iRGD in the concentration range of 0–50 μM, all being nearly 100% (Figure 2), indicating that iRGD, iRGE, or CG_7_C did not exert appreciable cytotoxic effects within the given concentration range.

### Cell uptake of iRGD *in vitro*

**FIGURE 3:**
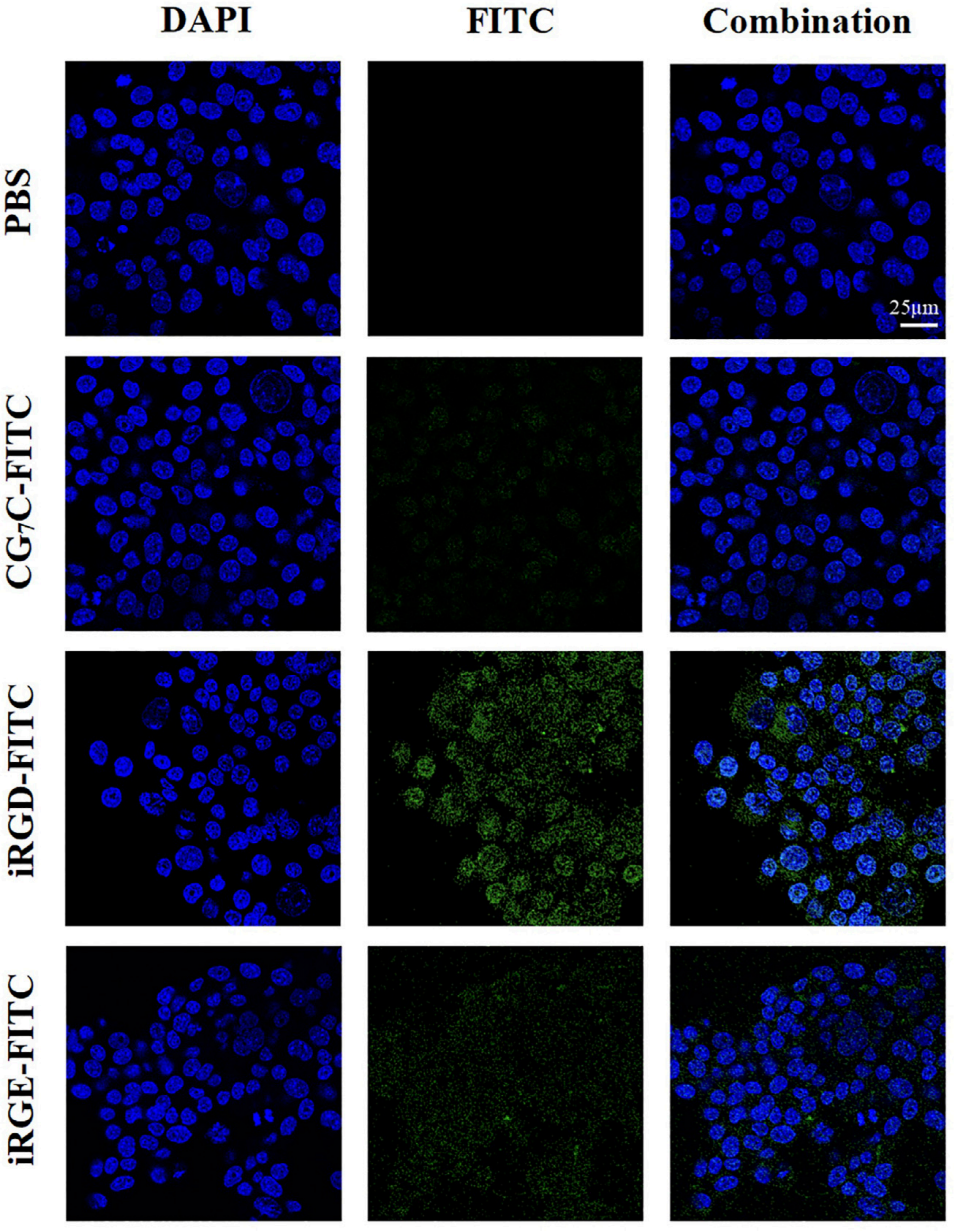
Confocal microscopy images of 4T1 cells incubated with CG_7_C-FITC, iRGD-FITC, or iRGE-FITC at a concentration of 10 μM for 4 h. The scale bar represents 25 μm for all panels.

**FIGURE 4:**
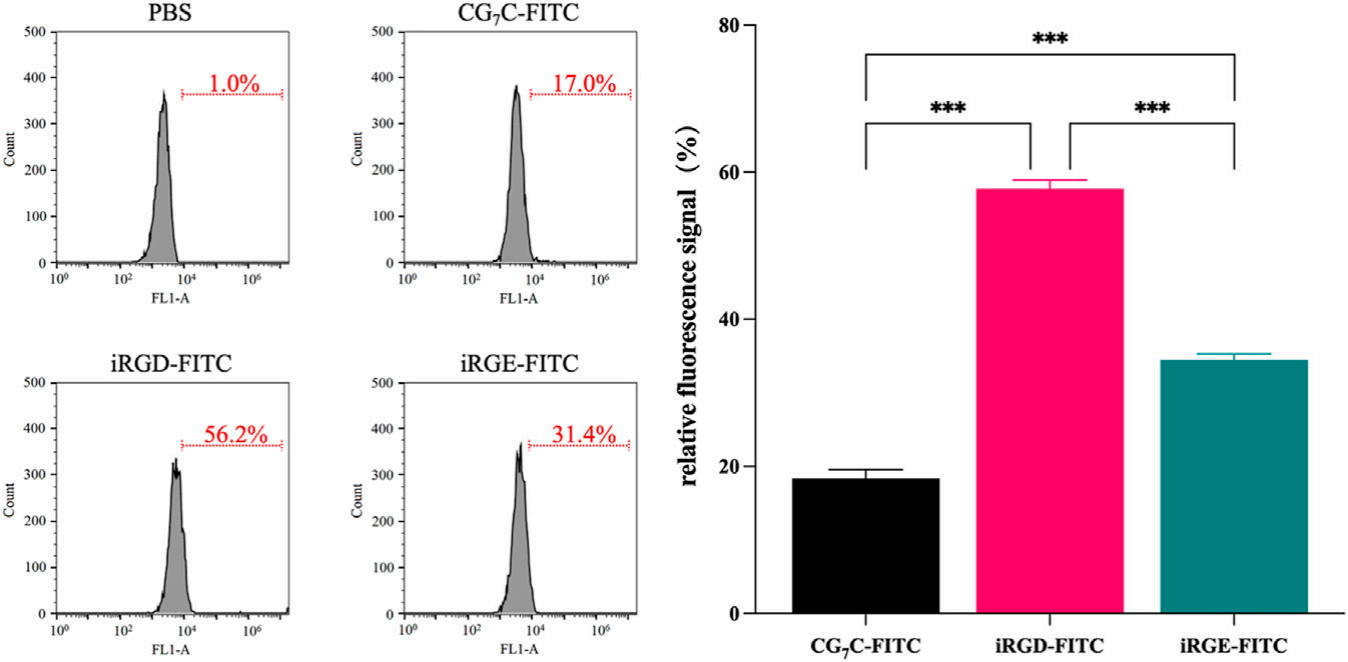
Cellular uptake and quantitative analysis of CG7C-FITC, iRGD-FITC, or iRGE-FITC by 4T1 cells were examined by flow cytometry at a concentration of 10 μM for 4 h.

Conjugating FITC with iRGD enables qualitative observation by confocal microscopy and quantitative detection by flow cytometry. For confocal microscopy, a more obvious FITC signal was observed in cells incubated with iRGD-FITC for 4 h compared to cells treated with iRGE-FITC and CG_7_C-FITC (Figure 3), confirming that the uptake efficiency of iRGD-FITC by the 4T1 cells is better than iRGE-FITC and CG_7_C-FITC. We then compared the specific cellular uptakes of iRGD, iRGE, and CG_7_C in 4T1 cells using flow cytometry. At a concentration of 10 μM for 4 h, the fluorescence intensity of 4T1 cells treated with iRGD-FITC was significantly higher than that of those treated with iRGE-FITC and CG_7_C-FITC and the fluorescence intensity of 4T1 cells treated with iRGE-FITC was higher than that of those treated with CG_7_C-FITC (Figure 4).

### *Ex vivo* fluorescent imaging

**FIGURE 5:**
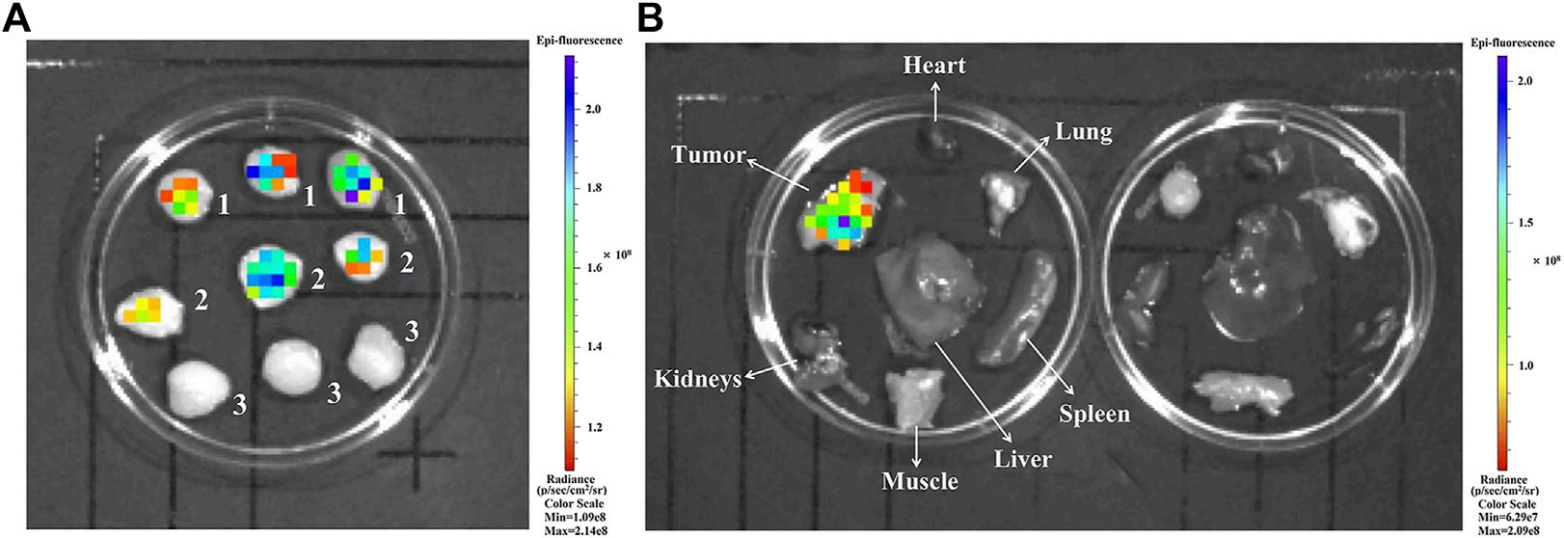
**(A)** *Ex vivo* fluorescence images of tumors at 1 h post-injection of (1) iRGD-FITC, (2) iRGE-FITC, and (3) CG_7_C-FITC; **(B)** *Ex vivo* fluorescence images of major organs and tumors at 2 h post-injection of iRGD-FITC (left) and iRGE-FITC (right).

To investigate the tumor targeting ability of iRGD *in vivo*, iRGD-FITC, iRGE-FITC, and CG_7_C-FITC were intravenously injected into mice bearing 4T1 tumors for *ex vivo* fluorescent imaging. The results showed no obvious tumor accumulation in the CG_7_C-FITC group at 1 h post-injection, which is in agreement with the literature (Feng et al., 2014), while iRGD-FITC and iRGE-FITC showed strong fluorescence intensity in tumors with no significant difference (Figure 5A). Additionally, the difference in fluorescence intensity in the tumors between iRGD-FITC and iRGE-FITC was compared at 2 h post-injection. The results indicated that iRGD-FITC showed a higher accumulation in tumors than iRGE-FITC under the same conditions (Figure 5B). Based on the imaging data, iRGD-FITC showed the best capability in tumor penetration and retention as compared to iRGE-FITC and CG_7_C-FITC.

### Single-positron emission computed tomography imaging and immunohistochemistry

**FIGURE 6:**
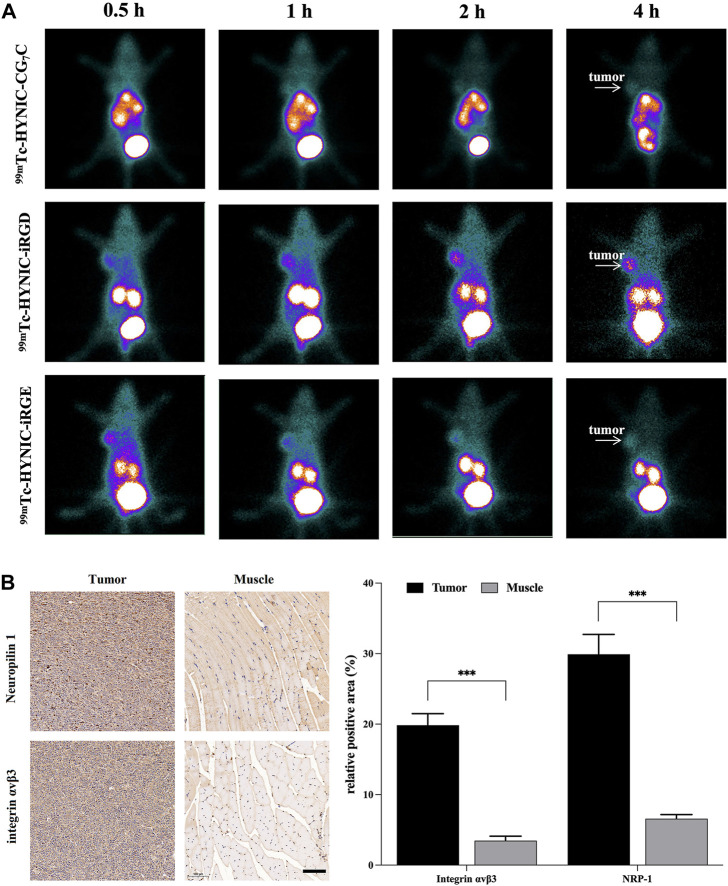
**(A)** SPECT images of 4T1 tumor-bearing nude mice at different time points after intravenous injection of ^99m^Tc-HYNIC-iRGD, ^99m^Tc-HYNIC-iRGE, and ^99m^Tc-HYNIC-CG_7_C; **(B)** Immunohistochemistry images and quantitative analysis of integrin αvβ3 and neuropilin-1 in tumor and muscle. The scale bar represents 100 µm for all panels.

To investigate the feasibility of ^99m^Tc-HYNIC-iRGD as a probe for TNBC imaging *in vivo*, we injected ^99m^Tc-labeled peptides *via* the tail vein into nude mice bearing 4T1 tumors. As shown in Figure 6A, high radioactivity was observed in the bladder and kidneys of mice treated with ^99m^Tc-HYNIC-iRGD, ^99m^Tc-HYNIC-iRGE, and ^99m^Tc-HYNIC-CG_7_C at each time point, suggesting that all peptides could be cleared through the urinary system. Although distinct tumor accumulation was observed at 0.5 h post-injection for ^99m^Tc-HYNIC-iRGD and ^99m^Tc-HYNIC-iRGE, the SPECT signal intensity of ^99m^Tc-HYNIC-iRGE in tumor gradually faded with time. Meanwhile, an increase in tumor accumulation of radioactivity was found in the mice treated with ^99m^Tc-HYNIC-iRGD, suggesting better tumor retention. As a negative control, ^99m^Tc-HYNIC-CG_7_C showed no obvious radioactivity distribution in the tumors during the study period, as expected. After SPECT imaging, integrin α_v_β_3_ and NRP-1 expression levels in tumors were confirmed by immunohistochemistry, and muscle tissues were used as negative controls. As shown in Figure 6B, the expression levels of integrin αvβ3 and NRP-1 in tumors were much higher than in muscle tissues. These results were consistent with TNBC molecular imaging based on iRGD targeting integrin αvβ3 and NRP-1.

### Biodistribution

**FIGURE 7:**
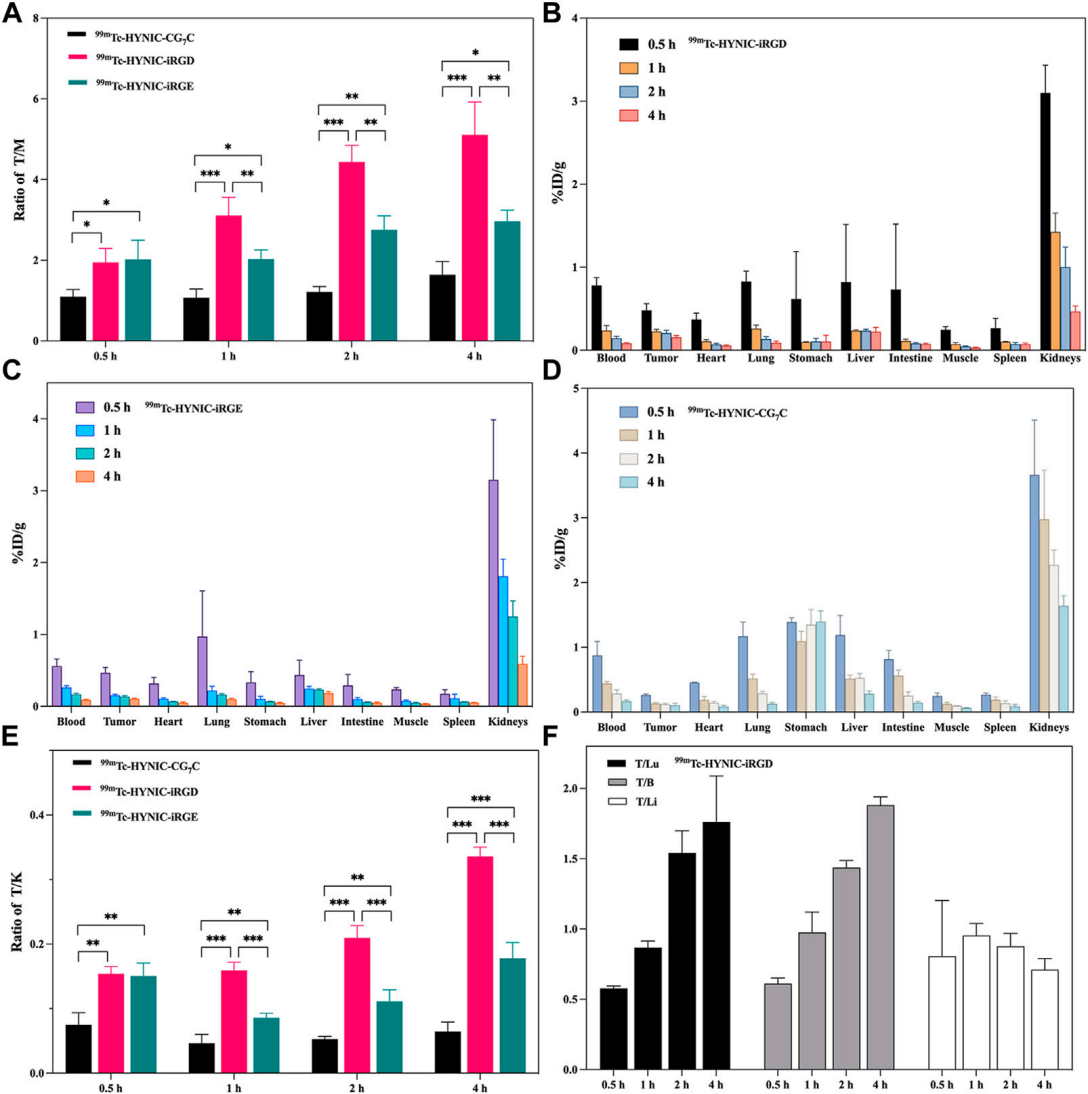
**(A)** The T/M ratios of ^99m^Tc-HYNIC-iRGD,^99m^Tc-HYNIC-iRGE, and ^99m^Tc-HYNIC-CG_7_C at 0.5, 1, 2, and 4 h. Biodistribution of **(B)** ^99m^Tc-HYNIC-iRGD, **(C)** ^99m^Tc-HYNIC-iRGE and **(D)** ^99m^Tc-HYNIC-CG_7_C at 0.5, 1, 2, and 4 h. **(E)** The T/K ratios of ^99m^Tc-HYNIC-iRGD, ^99m^Tc-HYNIC-iRGE, and ^99m^Tc-HYNIC-CG_7_C at 0.5, 1, 2, and 4 h; **(F)** The T/Lu, T/B, and T/Li ratios of ^99m^Tc-HYNIC-iRGD at 0.5, 1, 2, and 4 h.

The superior tumor imaging performance of iRGD was further verified by quantitatively analyzing the radioactivity of the main organs and tumors from 4T1 tumor-bearing mice treated with ^99m^Tc-HYNIC-iRGD, ^99m^Tc-HYNIC-iRGE, and ^99m^Tc-HYNIC-CG_7_C. The tumor-to-muscle (T/M) ratios at different time points are shown in Figure 7A. Although the T/M ratio increased with time, it was higher in mice treated with ^99m^Tc-HYNIC-iRGD than in those treated with ^99m^Tc-HYNIC-iRGE at each time point. Conversely, the T/M ratio in mice treated with ^99m^Tc-HYNIC-CG_7_C was stable at the range of 1.0–1.6. Consistent with the SPECT images, the T/M ratios of ^99m^Tc-HYNIC-iRGD and ^99m^Tc-HYNIC-iRGE at 0.5 h were comparable, while the difference between them was more and more conspicuous from 1–4 h. Moreover, the biodistribution in mice treated with ^99m^Tc-HYNIC-iRGD, ^99m^Tc-HYNIC-iRGE, or ^99m^Tc-HYNIC-CG_7_C from 0.5–4 h post-injection is shown in Figures 7B–D. Generally, there were no obvious differences between the groups. The main SPECT signal accumulated in the kidneys, with relatively low accumulation in other major organs, including the heart, lung, stomach, intestine, blood, liver, spleen, and muscle. Unlike ^99m^Tc-HYNIC-iRGD and ^99m^Tc-HYNIC-iRGE, ^99m^Tc-HYNIC-CG_7_C exhibited mild uptake and retention in the stomach. Notably, the tumor-to-kidney (T/K) ratio for ^99m^Tc-HYNIC-iRGD was much higher than for ^99m^Tc-HYNIC-iRGE and ^99m^Tc-HYNIC-CG_7_C, further confirming its superior tumor imaging performance (Figure 7E). Considering that the liver, lung, and bone are the most frequent metastatic sites of breast cancer, the tumor-to-blood (T/B) ratio is essential for evaluating the contrast effect, as blood-borne activity may reduce the imaging quality (Weigelt et al., 2005). Thus, we analyzed the T/B, tumor-to-liver (T/Li), and tumor-to-lung (T/Lu) ratios of ^99m^Tc-HYNIC-iRGD. As shown in Figure 7F, although the ratio of T/Li fluctuated between 0.7 and 1, indicating that the contrast between the tumor and liver was not very obvious, good contrast effects between the tumor and lung or blood were shown because the T/Lu and T/B ratios were as high as 1.9 and 1.8, respectively, at 4 h post-injection.

### Pharmacokinetics

**FIGURE 8:**
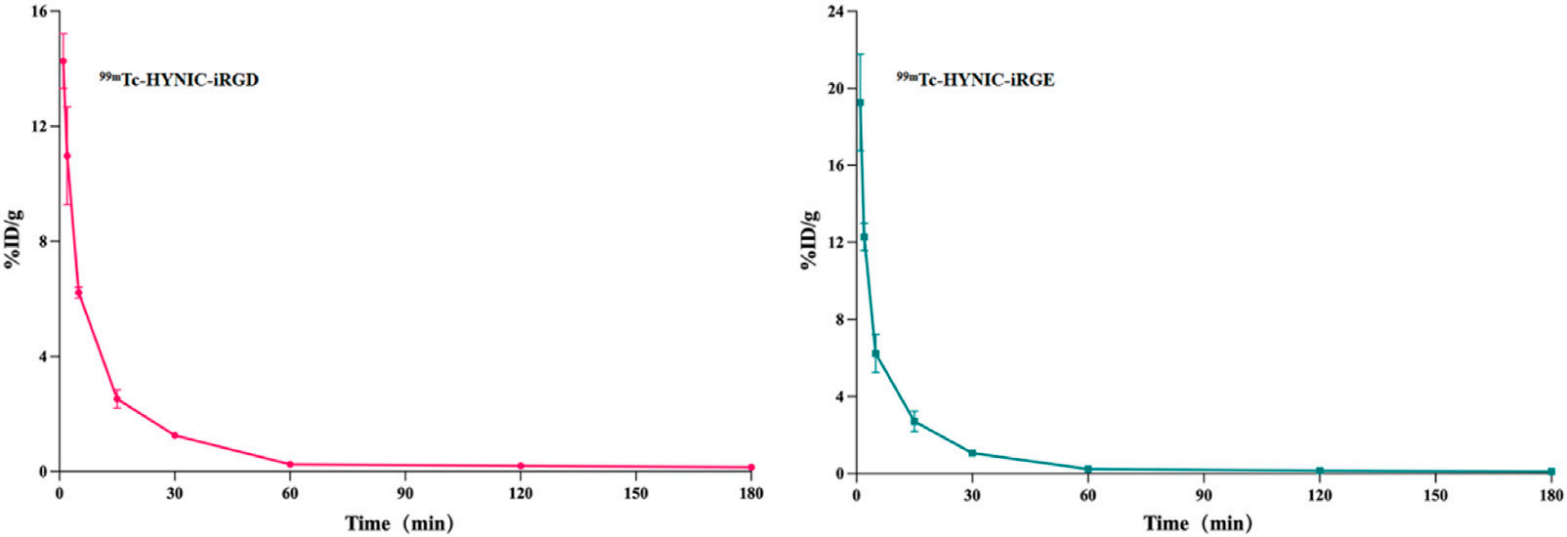
Radioactivity-time curve of ^99m^Tc-HYNIC-iRGD and ^99m^Tc-HYNIC-iRGE in normal mice.

The radioactivity-time curves of ^99m^Tc-HYNIC-iRGD and ^99m^Tc-HYNIC-iRGE are shown in Figure 8. Fast blood clearance was found within 0.5 h. The initial radioactivity levels of ^99m^Tc-HYNIC-iRGD and ^99m^Tc-HYNIC-iRGE in blood were 14.27 ± 0.95 %ID/g and 19.26 ± 2.52 %ID/g at 1 min and quickly fell to 1.26 ± 0.11 %ID/g and 1.06 ± 0.08 %ID/g at 30 min. Then, the radioactivity in blood was cleared at a slow rate, and approximately 0.1 %ID/g remained at 180 min. The distribution phase half-lives t_1/2_(α) of ^99m^Tc-HYNIC-iRGD and ^99m^Tc-HYNIC-iRGE were estimated to be 1.43 and 0.88 min, respectively, and their clear-phase half-lives t_1/2_(β) were 12.54 and 10.56 min, respectively. These data suggest that ^99m^Tc-HYNIC-iRGD had a longer circulation time than ^99m^Tc-HYNIC-iRGE *in vivo*.

## Discussion

Many patients with TNBC are already in advanced stages when they are newly diagnosed, leading to a higher rate of early recurrence and distant metastasis to the brain or lung compared to other breast cancer subtypes (Carey et al., 2010). Molecular imaging has great potential for the diagnosis and monitoring of early recurrence of breast cancer. In this study, the iRGD peptide was selected as an active targeting molecule for TNBC imaging because of its high binding affinity for integrins and NRP-1, which are overexpressed in TNBC cells. The iRGD peptide has been widely studied for tumor imaging using various imaging methods including MRI, ultrasound, optimal imaging, and positron emission tomography (Teesalu et al., 2009; Bohn et al., 2013). Previous studies have focused on imaging agents conjugated or modified with iRGD to improve imaging diagnostic effects compared with their non-iRGD-linked counterparts (Hou Dong Zuo et al., 2014; Cho et al., 2016; Yang et al., 2018; Moncelet et al., 2013). However, the superiority of radiolabeled iRGD has rarely been highlighted. In the present study, we prepared ^99m^Tc-labeled iRGD as a SPECT probe for TNBC imaging and used iRGE and CG_7_C as control peptides for different purposes. The CG_7_C peptide has poor tumor targeting ability owing to the lack of RGD and CendR motifs. The iRGE peptide, a non-integrin binding variant of iRGD contains only a cryptic CendR motif to validate the role of the RGD motif in targeting ability (Sugahara et al., 2009).

The iRGD peptide has been used for cancer therapy in several studies (Houdong Zuo 2019). A recent study demonstrated that iRGD-modified nanoworms significantly affected tumor progression in the early stages of metastasis, which might be regulated by binding NRP-1, with no or minor effect on primary tumors, whereas free iRGD peptide did not significantly inhibit primary and metastatic tumors (Hamilton et al., 2015). Hence, the potential cytotoxic effects of iRGD were first assessed in this study, and no obvious cytotoxicity was observed in 4T1 tumor cells. The ability of iRGD to target 4T1 cells was evaluated *in vitro* using confocal microscopy and flow cytometry. The data demonstrated that iRGD-FITC could better target 4T1 cells and penetrate tumor tissues than iRGE-FITC and CG_7_C-FITC. Notably, the fluorescence intensity of 4T1 cells treated with iRGE-FITC was higher than CG_7_C-FITC, suggesting that iRGE could also target 4T1 cells but less effectively than iRGD. The main reason is that the integrin binding motif (RGD motif) of iRGD facilitates the concentration of peptides on the 4T1 cell surface to expose the CendR motif for further internalization into tumor cells, while the lack of an RGD motif leads to the failure of iRGE to initially recruit to the surface of tumor cells, resulting in a weaker targeting ability than iRGD. This was also verified by *ex vivo* fluorescence imaging. The iRGD-FITC not only showed higher tumor accumulation but also a slower clearance from tumor than iRGE-FITC, supporting its better targeting ability to 4T1 cells.

HYNIC is a frequently used bifunctional chelator in technetium chemistry because of the easy modification property and high labeling efficiency of the labeled biomolecules (Meszaros et al., 2011; Kręcisz et al., 2021). In this study, we could readily label the HYNIC-modified peptides with ^99m^Tc using tricine and EDDA as co-ligands. Unsurprisingly, the formed ^99m^Tc-labeled peptides had high RCPs and stability under different conditions. SPECT imaging of 4T1 tumor-bearing mice injected with ^99m^Tc-HYNIC-iRGD displayed longer tumor retention time, resulting in better image quality than ^99m^Tc-HYNIC-iRGE. This is because integrin αvβ3 is highly expressed on the surface of tumor neovascular endothelial cells and 4T1 tumor cells but not in normal tissues, which enables better accumulation of iRGD in tumor tissues *in vivo* (Xin et al., 2016), whereas iRGE only targets tumor tissues *via* the CendR pathway through NRP-1, which is expressed both in tumor cells and normal tissues (Mamluk et al., 2002; Gu et al., 2003; Ellis 2006). Moreover, the vascular endothelium is the gateway to the tumor for imaging agents, but NRP-1 is expressed in all vessels, not just tumor vessels (Teesalu et al., 2009), resulting in weaker specificity for iRGE than for iRGD.

In summary, the reasons for ^99m^Tc-HYNIC-iRGD having better tumor targeting ability and retention than ^99m^Tc-HYNIC-iRGE are mainly attributed to the following two aspects. First, the RGD motif improves the efficiency of iRGD in reaching the surface of tumor cells, and the proteolytic process only occurs on the cell surface, which is crucial for further cellular internalization. Second, iRGD can actively bind to integrin αvβ3 for NRP-1 binding, and iRGE only targets tumor cells through NRP-1 receptor. Integrin αvβ3 is highly expressed in tumor tissues, whereas NRP-1 is widely expressed in both tumor and normal tissues, which weakens the tumor specificity of iRGE to a certain extent. Further research is required to elucidate the detailed mechanism. Similar to iRGE, the targeting peptide CLKADKAKC (CK3) contains a potential CendR motif and has been studied for SPECT imaging of breast cancer, but no comparative analysis has been performed for the difference in tumor targeting ability between CK3 and iRGD (Feng et al., 2014). Our results demonstrated that iRGD with the RGD motif and CendR motif showed better tumor targeting specificity than iRGE with only the CendR motif.

The biodistribution properties of ^99m^Tc-labeled peptides were studied and compared. The main radioactivity was observed in the kidneys owing to the high hydrophilicity of peptides after HYNIC modification, which allows the kidney to be the primary excretory organ. The sensitivity of radionuclide imaging depends on its contrast, which in turn depends on the ratio of radioactivity in the tumors and surrounding tissues (Tolmachev et al., 2021). The higher ratios of T/M and T/K of ^99m^Tc-HYNIC-iRGD were consistent with SPECT imaging, which showed conspicuity of tumor imaging and further verified its better tumor targeting ability than ^99m^Tc-HYNIC-iRGE and ^99m^Tc-HYNIC-CG_7_C. The high T/Lu and T/B ratios at 4 h post-injection also endowed ^99m^Tc-HYNIC-iRGD with sensitivity in detecting lung metastasis and blood-borne activity in TNBC. However, the contrast effect between the tumor and liver was not obvious, which may not be helpful in detecting liver metastasis of TNBC. The pharmacokinetic data revealed that ^99m^Tc-HYNIC-iRGD had a longer blood circulation time than ^99m^Tc-HYNIC-iRGE *in vivo,* which may be attributed to the better retention of iRGD in tumor tissue than iRGE. Considering the satisfactory imaging performance of ^99m^Tc-HYNIC-iRGD in 4T1 tumor-bearing mice, it holds the potential to improve TNBC diagnosis. However, its radiation dosimetry is unclear, and its extended applications in TNBC, such as monitoring therapeutic effects, are also uncertain. Further studies are required to address these issues.

## Conclusion

In this study, we synthesized ^99m^Tc-HYNIC-iRGD as a SPECT probe and evaluated its feasibility in TNBC imaging. The iRGD peptide exhibited excellent biocompatibility and specificity for TNBC cells *in vitro*. The HYNIC-modified iRGD was readily labeled with ^99m^Tc using a simple method. The synthesized ^99m^Tc-HYNIC-iRGD exhibited high RCP and stability *in vitro*. SPECT imaging demonstrated preferential accumulation of ^99m^Tc-HYNIC-iRGD in the tumors of a TNBC mouse model with fast blood clearance and favorable biodistribution. Our findings indicate that this active-targeted strategy has great potential to be developed as a new tool for TNBC imaging.

## Data Availability

The original contributions presented in the study are included in the article/supplementary material, further inquiries can be directed to the corresponding authors.
